# Effects of IV Fluids in Dogs and Cats With Kidney Failure

**DOI:** 10.3389/fvets.2021.659960

**Published:** 2021-04-20

**Authors:** Cathy Langston, Daniel Gordon

**Affiliations:** The Ohio State University College of Veterinary Medicine, Columbus, OH, United States

**Keywords:** renal, kidney, acute kidney injury, chronic kidney disease, oliguria, anuria, polyuria

## Abstract

Intravenous fluid therapy has long been the mainstay of treatment of kidney disease, including acute kidney injury and uremic crisis associated with chronic kidney disease. Careful management of fluid dose is critical, as animals with kidney disease may have marked derangements in their ability to regulate fluid homeostasis and acid-base status. Understanding of the physiology of renal fluid handling is necessary, along with repeated attention to parameters of fluid status, electrolytes, and acid-base balance, to achieve optimal hydration status and avoid further damage or decrease in function from dehydration or overhydration.

## Introduction

Fluid therapy is the most commonly administered intravenous treatment for hospitalized veterinary patients. Adequate fluid resuscitation is essential for the restoration of cardiac output, systemic blood pressure, and renal perfusion. Achieving an appropriate level of volume management requires knowledge of underlying pathophysiology, evaluation of volume status, selection of appropriate solution for volume repletion and maintenance, and modulation of the tissue perfusion (1).

Recently, Hoste et al. (2) proposed four distinct phases (ROSE) of intravenous fluid therapy for humans with critical illness: (R) resuscitation, (O) optimization, (S) stabilization, (E) evacuation. In overview, the *resuscitation phase* anticipates an escalation of fluid therapy in patients with life-threatening shock (low blood pressure, signs of impaired perfusion, or both) and is characterized by the use of *fluid bolus* therapy (rapid infusion to correct hypotensive shock; ~10 ml/kg balanced isotonic crystalloid over 15 min; typically not exceeding a total of three boluses). When transitioning to the *optimization phase*, the patient should no longer be in immediate life-threatening danger, but in a stage of compensatory shock and high risk for decompensation. At this point, additional fluid therapy is given more cautiously with the intent of optimizing cardiac function and improving tissue perfusion. The goal is to administer the volume necessary to alleviate organ dysfunction. *Fluid challenges*, defined as a small volume over short period of time (~5 ml/kg over 20 min), are typically given in this phase to test the effects of delivering more volume (3). Some patients may bypass the *resuscitation phase* (with no evidence of hypotension) and present to the hospital in a compensated state. In these first two phases, compensatory neuroendocrine reflexes and possible renal dysfunction result in sodium and water retention, contributing to a positive fluid balance. Progression to the *stabilization phase* encompasses a point at which the patient is at steady state without signs of shock. Fluid therapy in this phase is used for ongoing maintenance losses (renal, gastrointestinal, insensible); as well as rehydration if the patient is experiencing ongoing losses due to unresolved pathology. Patients may experience temporary deterioration and revert back to the *optimization phase* if faced with a newly developed infection or organ dysfunction. Lastly, the *evacuation phase* is characterized by the removal of fluids and promotes a negative fluid balance (2, 4). This may need to be achieved with low doses of loop-diuretics (authors suggest 0.25–0.5 mg/kg IV). The physiological response to fluids and underlying conditions are dynamic over time, thus fluid administration should be based on repeated assessment of cumulative fluid balance and hemodynamic status. Generally, hemodynamic status can be assessed using dynamic testing to evaluate fluid responsiveness (5).

Interest in intravenous fluid therapy and its side effects has increased in recent years. Many trials have investigated the colloid/crystalloid debate, while others have provided guidance on selecting the optimal crystalloid solution (6). In addition, the role of chloride and its detrimental effect on kidney function has garnered much attention (7–10). Much of the research has focused on fluid therapy in the resuscitation phase, in which people receive large amounts of fluid over a short period of time. In reality, hospitalized people receive fluids and electrolytes for other reasons including maintenance therapy, medications, and replacement for existing or ongoing losses. The cumulative fluid delivery frequently exceeds fluid loss, leading to a net positive fluid balance or “fluid creep.” With this principle in place, additional considerations need to be made for renal patients with altered sodium handling.

## Pathophysiology

The primary function of the kidney is to maintain a stable extracellular compartment by selective retention/elimination of water, electrolytes, and other solutes. This is achieved via filtration of the circulating blood into ultrafiltrate from the plasma, selective reabsorption from tubular fluid into peritubular capillary blood, and selective secretion from peritubular capillary blood into the tubular fluid. Glomerular filtration (*net ultrafiltration pressure*) is determined by the difference in hydrostatic and oncotic pressure gradients between the glomerular plasma and the filtrate in Bowman space. Each nephron has its own single-nephron glomerular filtration rate (SNGFR), and the sum of the SNGFRs of the functioning nephrons make up the total glomerular filtration rate (GFR). Maintaining a normal GFR is dependent on adequate renal perfusion.

Acute kidney injury (AKI) is defined as a rapid decline in GFR through the measurement of serum creatinine and urine output. There are inherent challenges with this definition as creatinine is poorly correlated with GFR at low levels of renal dysfunction (11). Clinically, AKI is grouped into three etiologies: pre-renal, renal, and post-renal. Pre-renal azotemia is characterized by a decrease in GFR due to a decrease in renal perfusion pressure without damage to the renal parenchyma (12). Post-renal causes of AKI are characterized by acute obstruction of urinary flow, leading to increases in intratubular pressure, impaired renal blood flow and inflammatory processes, and decreased GFR (13). Renal azotemia is associated with a sudden onset of renal parenchymal injury, characterized by the kidneys inability to meet excretory, metabolic, and endocrine demands of the body. AKI represents a continuum of renal injury from mild, clinically inapparent nephron loss to severe acute renal failure requiring renal replacement therapy. The International Renal Interest Society (IRIS) AKI Grading scheme has been developed in veterinary medicine to determine appropriate grading in animals with AKI (14).

Intrinsic renal etiologies can be challenging to evaluate due to a wide variety of injuries that can occur to the kidney. In general, renal damage can be divided into four major structural groupings: the tubules, glomeruli, interstitium, and intra-renal blood vessels. Tubular damage can arise from either ischemic injury (decreased renal perfusion) or nephrotoxic compounds (exogenous and endogenous). Severe acute glomerulonephritis secondary to immune-complex disease causes glomerular damage. Interstitial damage can result from acute interstitial nephritis secondary to medications or infectious etiologies (leptospirosis, pyelonephritis, etc.). Lastly, vascular damage can occur secondary to injury to intra-renal vessels (thrombosis, hypertension, etc.), subsequently decreasing renal perfusion and diminishing GFR (15). Ischemia and nephrotoxic agents (e.g., certain drugs, plants, ethylene glycol, etc.) account for the majority of renal injury in human medicine as well as veterinary medicine (16).

Renal tubular injury is associated with a decrease in GFR and is divided into different phases of AKI, which are directly related to the cellular events that occur during the injury and recovery process. The *initiation phase* occurs when renal blood flow decreases, resulting in cellular ATP depletion and subsequent tubular epithelial cell injury. These changes alter the ability of tubular epithelial cells and vascular endothelial cells to maintain normal renal function (reabsorption and secretion), as well as up-regulate a variety of chemokines and cytokines to initiate an inflammatory cascade (17). The *extension phase* is characterized by continued hypoxia following the initial ischemic event and an inflammatory response, most prominent in the outer medullary region of the kidney. During this phase, vascular endothelial damage likely plays a key role in the continued ischemia of the tubular epithelium, as well as the inflammatory response. Cells in the outer medullary region undergo injury and death via necrosis and apoptosis (18). The cellular injury in this region leads to the continual reduction in GFR, whereas cells of the proximal tubule in the outer cortex, where blood flow has returned, undergo cellular repair and improve morphologically (19). The *maintenance phase* consists of cells undergoing repair, migration, apoptosis, and proliferation to reestablish and maintain cellular and tubular integrity. The GFR usually remains stable at the level determined by the severity of the initial event. Cellular repair and reorganization results in slowly improving cellular function, setting the stage for improvement in organ function. Blood flow returns toward normal and epithelial cells establish intracellular and intercellular homeostasis (19). During the *recovery phase*, cellular differentiation continues, epithelial polarity is re-established, and normal cellular and organ function returns (20). Alternatively, renal repair can be maladaptive with inflammation, fibrosis, and vascular rarefaction leading to persistent cell and tissue malfunction and eventually chronic kidney disease (21). AKI and chronic kidney disease (CKD) are increasingly recognized as related entities representing a continuum of disease (22).

The assessment of renal recovery is controversial with a lack of clear, defined parameters. Despite the limitations, the most obvious definition of full recovery from AKI is the absence of AKI criteria. Partial recovery is defined as a fall in AKI grade. Recovery may occur early, after the insult (within 7 days), or later, during the proposed period of acute kidney disease (AKD), which is defined as AKI persisting for 7–90 days (23). Lack of complete recovery in the first 90 days is defined as CKD.

Disruption of normal renal function impairs maintenance of homeostasis. A normochloremic high anion gap metabolic acidosis is common, due to inadequate excretion of organic and inorganic acids such as phosphate and sulfate. Hyperkalemia is also common when urine production is low and distal tubular secretion of potassium is impaired. Conversely, hypokalemia may develop with polyuria, as the high urine flow through the distal tubule enhances potassium secretion by maintaining a concentration gradient between the tubular cells and the ultrafiltrate in the tubule lumen. Disorders of sodium are variable and depend on the degree of impairment of sodium reabsorption relative to water excretion. Because of impaired filtration and thus excretion of phosphate, leading to hyperphosphatemia, an ionized hypocalemia may develop acutely.

## Aims of Fluid Resuscitation

The physiological rationale for fluid administration in AKI and CKD is to optimize intravascular circulating volume, increase cardiac output and perfusion pressure, with the aim of improving renal blood flow, renal oxygen supply, and GFR (Table 1). Hypotension is a strong risk factor for AKI, yet preserving systemic arterial pressure alone is not adequate for renal perfusion. Renal tissue perfusion relies on the pressure in the post-glomerular arterioles, which is often much lower than the systemic MAP (21). Conversely, fluid resuscitation beyond correction of hypovolemia does not increase the chances of renal recovery. Excessive fluid administration has been associated with the development of AKI, secondary to intrarenal compartment syndrome and venous congestion, attributed to the kidneys being encapsulated organs (24, 25). It is imperative to understand the physiological response to fluids and underlying condition related to AKI are dynamic, thus fluid administration should be based on repeated assessments of the patient and relevant biomarkers. Overall fluid and hemodynamic status, using dynamic tests of fluid responsiveness are utilized to determine the need for additional fluid therapy (5). The clinician is forced to walk a tight rope between too few fluids, which can lead to further progression of AKI from ongoing renal ischemia, and too much fluids leading to systemic complications and organ dysfunction.

**Table 1:** Goals of fluid resuscitation in AKI.

•Optimize intravascular circulating volume •Increase cardiac output •Improve perfusion pressure •Improve renal blood flow, renal oxygen supply, and GFR •Dislodge debris and casts obstructing tubules

## Fluid Types

Fluids are differentiated into crystalloids and colloids. Disregarding the impact of fluid overload, there is growing evidence that renal function is affected by the type of fluid and certain fluids are associated with an increased risk of AKI (24, 26, 27).

## Isotonic Crystalloids

Isotonic crystalloids are the accepted first-line IV fluid in most ICU patients. There is ongoing research as to which isotonic crystalloid, 0.9% NaCl vs. a buffered crystalloid (LRS, Plasma-Lyte, Hartmann's), is appropriate in people at risk for AKI. The impact of chloride concentration on renal function has been researched in a Greyhound model, showing that increasing plasma chloride levels produce progressive renal vasoconstriction and decreased GFR. These effects appeared to be related to tubular chloride reabsorption (28). Both animal research and studies in healthy human volunteers suggest that hyperchloremia may lead to renal vasoconstriction, reduction in renal cortical tissue perfusion and glomerular filtration, along with longer periods of fluid retention compared with buffered crystalloids (29).

Clinical trials comparing various crystalloid fluids in critically ill people at risk of AKI have produced conflicting results (7, 9, 10, 30). The SMART and SALT-ED trials are the largest randomized controlled trials comparing 0.9% NaCl and buffered crystalloids. Both found a significant reduction in the risk of major kidney events within 30 days, in the group treated with buffered solutions (9, 10). Despite these findings, the optimal fluid in people at risk of AKI has not been identified. It is of the authors opinion that balanced crystalloids should be used for resuscitation and replacement therapy. Dextrose-containing solutions should not be used as a replacement solution unless the patient is hypoglycemic or a neonate/infant.

## Hypotonic Crystalloids

Maintenance solutions (hypotonic crystalloids) are administered to meet the patient's basal requirements of water and electrolytes. Specific maintenance solutions are commercially available but are far from ideal. Studies in people have shown over the course of an ICU admission, a substantial amount of daily fluid volume is delivered with maintenance/replacement fluids, medications, and nutrition compared to resuscitation fluids (31). Generally, the overall sodium and chloride load administered with maintenance and replacement fluids is not taken into consideration, even though these fluids account for a large portion of the daily fluid volume in these patients. There is debate whether isotonic or hypotonic solutions should be used for maintenance fluid therapy. Much of the data in children has showed a possible risk of hyponatremia (32, 33). Studies in human adults indicate a more positive fluid balance and decreased urine output with isotonic solutions compared to hypotonic solutions (34, 35). These studies bring to light the concept of “fluid creep” secondary to high daily sodium administration with isotonic crystalloids leading to fluid retention. Further studies in patients with AKI are needed to make a recommendation, but in theory these patients with altered sodium handling and higher chloride concentrations reaching the macula densa would benefit from a hypotonic solution in the *stabilization phase*. At this stage, the authors preferentially use hypotonic solutions to reduce sodium load in animals with renal dysfunction. Monitoring the animal's electrolytes and acid-base status frequently is essential for prescribing the appropriate fluid during the resuscitation and early stabilization phase.

## Synthetic Colloids

In human medicine, the administration of synthetic colloids has been linked with the development of acute kidney injury (AKI) and the need for renal replacement therapy (RRT) in ICU patients, especially those with sepsis (27, 36). Findings from meta-analyses suggest this may depend on patient cohort, but did confirm a higher risk of AKI and conflicting reports on mortality (37). In veterinary medicine, there is inconclusive evidence on the development of AKI in dogs and cats treated with hydroxyethyl starches (HES). The majority of the studies are retrospective, with inconsistent definitions of AKI (38–41). Extrapolating from human medicine, in most clinical situations, the risks outweigh the benefits and alternative volume replacement therapies should be used in place of HES.

## Natural Colloids

Albumin is a natural colloid, specifically studied in resuscitation, that has not been shown to have consistent survival benefit. Although, albumin is considered safe in people at risk or with established AKI, studies are needed to assess potential benefits (24, 26, 42). The most recent Surviving Sepsis Campaign recommendations state that albumin in addition to crystalloids may be considered in limited quantities for early resuscitation and subsequent intravascular volume replacement in people with sepsis or septic shock (43). HES should be avoided in this patient population, thus other colloids (FFP and albumin) may be considered in limited quantities. There are no studies in veterinary medicine regarding the use of FFP or albumin in dogs or cats with renal dysfunction.

## Fluid Dose

There are few evidence-based studies evaluating the dose response of intravenous fluids in animals with kidney dysfunction. Standard formulas for what is commonly called a maintenance intravenous fluid rate make two presumptions, namely that animals have similar evaporative losses and that urine output is “average” (1–2 mL/kg/h). In patients with renal dysfunction, standard maintenance formulas do not apply and fluid requirements must be assessed on an individual basis. Evaporative losses in dogs and cats are predominantly through the respiratory tract, as perspiration is generally negligible, and salivary and fecal losses are generally not accounted for when determining fluid balance. Respiratory losses can be quite dissimilar and are impacted by environmental temperature and humidity, and by patient activity level. In one study of healthy dogs, evaporative losses varied from 8.1 to 75.7 ml/kg/day, with a mean of 27 mL/kg/day (44–46). Barking and panting increased evaporative loss compared to losses in quiet dogs. In cats, insensible loss ranged from 12.4 to 29 ml/kg/day in different studies (44, 47, 48). Insensible loss is generally considered to be 22 ml/kg/day, regardless of demeanor. In the animal with kidney disease, urine volume is highly variable, ranging from extreme polyuria to anuria, necessitating adjustment of fluid administration rates based on the specific, and changing individual animal fluid requirements. Accurate urine output quantification is obtained through the placement of a urinary catheter. Alternatively, if urine cannot be accurately measured due to the inability to place a urinary catheter, a towel or diaper with a pre-specified weight can be placed in the cage. The difference in the weight of the towel or diaper when the animal urinates is approximately equal to the urine produced (1 g increase in weight = 1 mL urine).

Westgren et al. evaluated 11 dogs with suspected kidney disease using scintigraphy to measure GFR before and after a 15 ml/kg intravenous bolus of crystalloid fluid (49). The GFR appeared to increase 31% after the fluid bolus when measurements were calculated based on weight. However, when GFR was evaluated based on plasma volume, there was no change in GFR after fluid therapy. Given the effects that a fluid bolus can have on renal dynamics, this new method of calculating GFR suggests that fluid therapy does not acutely increase GFR, although the technique needs further validation.

In one study of systemically healthy dogs undergoing an orthopedic surgery and receiving standard intraoperative fluid rates (10 ml/kg/h of lactated Ringers solution), the median urine output was 0.46 mL/kg/h (50). GFR was stable during anesthesia. These dogs gained a median of 1.1 L of fluid (3.4% of pre-anesthesia body weight) over the 4-h anesthetic period, which was mostly distributed to the extracellular space, despite having normal kidney function. The neurohumoral effects of anesthesia appear to be able to affect renal hemodynamics even in normal animals and the effects of depth of anesthesia on urine output requires further investigation.

Brandstrup et al. compared goal directed therapy to a zero-balance approach in humans undergoing colorectal surgery (51). In the goal directed approach, fluid was administered to maintain near maximal stroke volume, whereas the zero-balance approach only replaced fluid losses. There was no difference in outcomes. Thus, a zero-balance approach appears safe and requires less invasive monitoring. Similar studies in clinical veterinary patients with kidney disease do not exist, although this approach merits investigation. Zero-balance fluid administration can be achieved by monitoring urine output and adjusting fluid dose accordingly (Table 2).

**Table 2:** Fluid dose guidelines.

•Resuscitation Phase: 10 ml/kg over 15 min, up to 3 boluses •Optimization Phase: 5 ml/kg over 20 min •Stabilization Phase^*^: Replace insensible loss at 22 ml/kg/day plus replacement of urine volume and additional loss (such as vomiting). The volume of medications and nutrition account for part of the fluid administered; the remaining volume is typically administered as a crystalloid fluid. •Evacuation Phase: Decrease fluid dose by 10–20% every 12–24 h and monitor for dehydration

* *Presumes any dehydration deficit was corrected during resuscitation and optimization phases. If volume overload already exists from prior fluid therapy, decrease fluid rate to allow correction of the overload over ~24 h*.

## Complications

**Figure 1:**
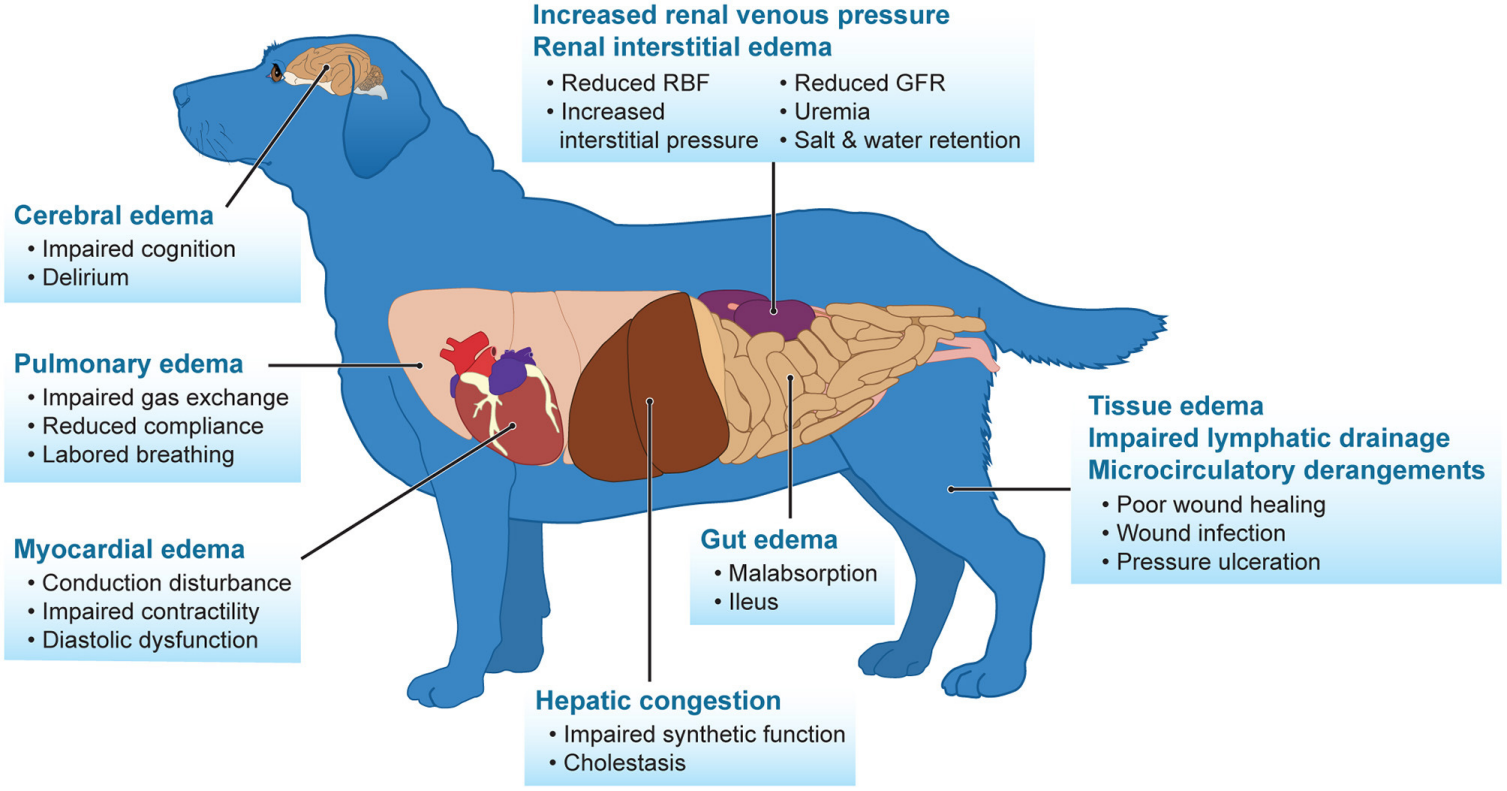
Fluid overload adversely impacts many body systems. Adapted from Prowle et al. (25).

Fluid overload is a commonly mentioned negative side effect of intravenous fluid therapy, especially in patients with initial renal dysfunction. It is well-documented in humans, and sparse evidence in veterinary medicine indicates that fluid overload contributes to progression of kidney dysfunction and increase in mortality (52–58). In addition, fluid overload has severe consequences affecting many different organs including the central nervous system, cardiovascular, pulmonary, hepatic, renal, and gastrointestinal tract (Figure 1) (4, 59). Excessive fluid administration is an iatrogenic complication that results in renal congestion, subsequently worsening renal perfusion and GFR due to interstitial edema. This is an area of harm seen in human and veterinary patients in attempts to “diuresis” the kidneys, ultimately leading to progressive renal dysfunction. Monitoring urine output in conscious animals is a sensitive and early marker for the detection of AKI, but also enhances the clinician's ability to track fluid overload and assess response to therapy. Cumulative fluid balance is the sum total of fluid accumulation over a set period of time and the percentage of fluid accumulation is defined by dividing the cumulative fluid balance by the patient's baseline body weight and multiplying by 100%. Diuretics remain a valid therapy for relieving symptoms and improving pathophysiological states of fluid overload, even in people with renal dysfunction (60–62). A urine output of 3–4 ml/kg/h after diuretic use has been shown to rarely cause intravascular volume depletion as intravascular refilling can equilibrate at that rate (25). With the limitations in detecting early signs of AKI due to the lack of active renal biomarkers in veterinary medicine, the authors recommend placing a urinary catheter in unstable cardiovascular, azotemic people to accurately monitor urine production. Because serum creatinine and oliguria are often late signs of significant AKI, a functional assessment of renal tubular function can indicate risk of AKI progression. Furosemide is a loop diuretic with ideal pharmacokinetic properties to assess tubular function. It is not effectively filtered by the glomerulus but is bound to serum proteins and gains access to the tubular lumen via active secretion in the proximal tubule. Once in the tubular lumen, furosemide inhibits active chloride transport throughout the ascending thick limb of Henle, preventing sodium reabsorption and inducing naturesis to increase urine flow (63). A furosemide stress test (1–1.5 mg/kg IV bolus) can be administered to oliguric patients that are assessed to be euvolemic or fluid overloaded to accurately determine AKI progression and need for renal replacement therapy (64). In human medicine, a 2-h urine cutoff of <200 mL produced the best sensitivity (87.1%) and specificity (84.1%) and was a predictor in progression of AKI (63). Unfortunately, this has not been studied in veterinary medicine, but extrapolating from humans, a response of 1.5 ml/kg/h roughly equates to the 200 mL over the 2 h time interval. Repeated administration of furosemide in a non-responsive patient can lead to adverse effects such as ototoxicity, which has been reported in humans.

The use of diuretics to increase urine output does not improve renal function. If renal replacement therapy is not available, diuretics may help control hyperkalemia by increasing distal tubular flow and allow more liberal administration of nutrition and medications with less risk of volume overload. Despite the widespread use of diuretics in human medicine, there are few guidelines pertaining to the dose and interval of administering furosemide. A recent meta-analysis in humans showed continuous furosemide was associated with greater diuretic effect in total urine output as compared to bolus dosing, although neither had any difference in mortality (65). The authors recommend the following doses (1–2 mg/kg IV q 6–8 h or 0.25–0.5 mg/kg/h IV) when administering furosemide. Hyperkalemia can be treated temporarily by causing an intracellular shift using regular insulin (0.5 u/kg IV with 1 g/kg IV dextrose). Beta-adrenergic agonists (e.g., terbutaline, albuterol) can be used as adjunctive therapy to shift potassium intracellularly but may require higher doses than need for bronchodilation. Bicarbonate therapy should be considered in dogs and cats with AKI and severe metabolic acidosis (i.e., pH <7.1 or serum bicarbonate <12 mEq/L). A significant beneficial effect has been shown in humans with AKI and severe metabolic acidosis to decrease the need for renal replacement therapy and overall mortality (66, 67).

## Clinical Context

Management of dogs and cats with AKI involves treating the underlying cause and providing time for the kidneys to repair themselves. Appropriate fluid therapy is paramount in the medical management of AKI, aiming to restore hydration and euvolemia. The preconceived notion to increase the fluid rate and force diuresis in animals with AKI must be absolutely avoided. This is especially true in animals with established oliguria or a relative oliguria, in which they are unable to appropriately respond by increasing urine production, thus developing generalized edema that directly exacerbates further organ dysfunction. If the animal is not able to remove the excess fluid due to lack of kidney function, renal replacement therapy is indicated to help facilitate fluid removal and achieve appropriate fluid balance. It is only once the animal has established a consistent urine output and advanced to the polyuric phase, that it becomes reasonable to increase the fluid rate to match urine output. Emphasis is placed on the importance of frequent patient assessments (cumulative fluid balance) to determine the fluid needs of these patients.

Fluids should be gradually tapered when hydration and urine production are restored, fluid “in” and urine “out” are matched, and the serum creatinine has plateaued (i.e., no further improvement at this stage). The authors recommend decreasing the fluid rate by 15–20% every 8 h, with the goal of discontinuing intravenous fluids in a 48 h period. It is not uncommon for animals with AKI to require a feeding tube (esophagostomy or gastrostomy) to not only administer proper nutrition and meet the demands of the hypercatabolic state, but also supplement enteral fluids in a markedly polyuric animal. The feeding tube allows the owner to play an integral role in the recovery phase of AKI as well as reduce the time and cost associated with hospitalization. A less physiologic option for maintaining hydration in an animal with AKI is the use of subcutaneous fluids. Subcutaneous fluids involve administration of large quantities of “salt water” (LRS, 0.9% NaCl), making this option less than ideal for animals with intrinsic renal dysfunction.

Consideration for fluid type (hypotonic and balanced isotonic) along with reduction in cumulative sodium load is more physiologic for dysfunctional kidneys. Additional studies are needed to characterize the urine chemistry of dogs and cats that present with AKI (68). We have become more cognizant to the negative effects of intravenous fluids and will continue to make new discoveries how the kidneys function in a dysfunctional state. As we gather more data in animals with renal injury, the question becomes truly which fluid type is recommended for these animals?

Unfortunately, accurate assessment of animal weight, which is used as a surrogate for fluid status, is challenging, as the body converts to a hypercatabolic state in established AKI and loss of muscle mass. Luckily, providing nutrition and water through an established feeding tube (E-tube, PEG, G-tube) is an ideal way to help manage the catabolism of muscle and daily fluid requirement. Ultimately, the best fluid therapy is one that maintains euvolemia, with the goal of delivering fluid in the most physiologic manner (via the GI tract).

## Author Contributions

CL and DG contributed to write the manuscript. All authors contributed to the article and approved the submitted version.

## Conflict of Interest

The authors declare that the research was conducted in the absence of any commercial or financial relationships that could be construed as a potential conflict of interest. The reviewer DC declared a shared affiliation with the authors to the handling editor at time of review.

## References

[1] Mehta RL Bouchard J. Controversies in acute kidney injury: effects of fluid overload on outcome. Controv Acute Kidney Inj. (2011) 174:200–11. 10.1159/000329410 21921625

[2] Hoste EA Maitland K Brudney CS Mehta R Vincent JL Yates D. Four phases of intravenous fluid therapy: a conceptual model†. Br J Anaesth. (2014) 113:740–7. 10.1093/bja/aeu300 25204700 PMC6863743

[3] Cecconi M Hofer C Teboul JL Pettila V Wilkman E Molnar Z. Fluid challenges in intensive care: the FENICE study: a global inception cohort study. Intensive Care Med. (2015) 41:1529–37. 10.1007/s00134-015-3850-x 26162676 PMC4550653

[4] Malbrain MLNG Van Regenmortel N Saugel B De Tavernier B Van Gaal P-J Joannes-Boyau O. Principles of fluid management and stewardship in septic shock: it is time to consider the four D's and the four phases of fluid therapy. Ann Intensive Care. (2018) 8:66. 10.1186/s13613-018-0402-x 29789983 PMC5964054

[5] Cecconi M Hernandez G Dunser M Antonelli M Baker T Bakker J. Fluid administration for acute circulatory dysfunction using basic monitoring: narrative review and expert panel recommendations from an ESICM task force. Intensive Care Med. (2019) 45:21–32. 10.1007/s00134-018-5415-2 30456467

[6] Semler MW Kellum JA. Balanced crystalloid solutions. Am J Respir Crit Care Med. (2019) 199:952–60. 10.1164/rccm.201809-1677CI 30407838 PMC6467313

[7] Yunos NM Bellomo R Hegarty C Story D Ho L Bailey M. Association between a chloride-liberal vs chloride-restrictive intravenous fluid administration strategy and kidney injury in critically ill adults. JAMA. (2012) 308:1566–72. 10.1001/jama.2012.13356 23073953

[8] Yunos NM Bellomo R Taylor DM Judkins S Kerr F Sutcliffe H. Renal effects of an emergency department chloride-restrictive intravenous fluid strategy in patients admitted to hospital for more than 48 hours: chloride-restrictive fluid strategy. Emerg Med Australas. (2017) 29:643–9. 10.1111/1742-6723.12821 28597505

[9] Semler MW Self WH Wanderer JP Ehrenfeld JM Wang L Byrne DW. Balanced Crystalloids versus Saline in Critically Ill Adults. N Engl J Med. (2018) 378:829–39. 10.1056/NEJMoa1711584 29485925 PMC5846085

[10] Self WH Semler MW Wanderer JP Wang L Byrne DW Collins SP. Balanced crystalloids versus saline in noncritically ill adults. N Engl J Med. (2018) 378:819–28. 10.1056/NEJMoa1711586 29485926 PMC5846618

[11] Ostermann M Bellomo R Burdmann EA Doi K Endre ZH Goldstein SL. Controversies in acute kidney injury: conclusions from a kidney disease improving global outcomes (KDIGO) conference. Kidney Int. (2020) 98:294–309. 10.1016/j.kint.2020.04.020 32709292 PMC8481001

[12] Badr KF Ichikawa I. Prerenal failure: a deleterious shift from renal compensation to decompensation. N Engl J Med. (1988) 319:623–9. 10.1056/NEJM198809083191007 3045546

[13] Hegarty NJ Young LS Kirwan CN O'Neill AJ Bouchier-Hayes DM Sweeney P. Nitric oxide in unilateral ureteral obstruction: Effect on regional renal blood flow. Kidney Int. (2001) 59:1059–65. 10.1046/j.1523-1755.2001.00589.x 11231361

[14] Cowgill LD Langston C. Acute kidney insufficiency. In: Nephrology and Urology of Small Animals (John Wiley & Sons, Ltd) (2011). p. 472–523. 10.1002/9781118785546.ch49

[15] Basile DP Anderson MD Sutton TA. Pathophysiology of acute kidney injury. Compr Physiol. (2012) 2:1303–53. 10.1002/cphy.c110041 23798302 PMC3919808

[16] Legatti SAM El Dib R Legatti E Botan AG Camargo SEA Agarwal A. Acute kidney injury in cats and dogs: a proportional meta-analysis of case series studies. PLoS ONE. (2018) 13:e0190772. 10.1371/journal.pone.0190772 29370180 PMC5784898

[17] Rabb H O'Meara YM Maderna P Coleman P Brady HR. Leukocytes, cell adhesion molecules and ischemic acute renal failure. Kidney Int. (1997) 51:1463–8. 10.1038/ki.1997.200 9150459

[18] Kelly KJ Plotkin Z Dagher PC. Guanosine supplementation reduces apoptosis and protects renal function in the setting of ischemic injury. J Clin Invest. (2001) 108:1291–8. 10.1172/JCI13018 11696573 PMC209442

[19] Sutton TA Fisher CJ Molitoris BA. Microvascular endothelial injury and dysfunction during ischemic acute renal failure. Kidney Int. (2002) 62:1539–49. 10.1046/j.1523-1755.2002.00631.x 12371954

[20] Nony PA Schnellmann RG. Mechanisms of renal cell repair and regeneration after acute renal failure. J Pharmacol Exp Ther. (2003) 304:905–12. 10.1124/jpet.102.035022 12604664

[21] Forni LG Darmon M Ostermann M Oudemans-van Straaten HM Pettilä V Prowle JR. Renal recovery after acute kidney injury. Intensive Care Med. (2017) 43:855–66. 10.1007/s00134-017-4809-x 28466146 PMC5487594

[22] Cowgill LD Polzin DJ Elliott J Nabity MB Segev G Grauer GF. Is progressive chronic kidney disease a slow acute kidney injury? Vet Clin North Am Small Anim Pract. (2016) 46:995–1013. 10.1016/j.cvsm.2016.06.001 27593574

[23] Chawla LS Bellomo R Bihorac A Goldstein SL Siew ED Bagshaw SM. Acute kidney disease and renal recovery: consensus report of the acute disease quality initiative (ADQI) 16 workgroup. Nat Rev Nephrol. (2017) 13:241–57. 10.1038/nrneph.2017.2 28239173

[24] Finfer S Myburgh J Bellomo R. Intravenous fluid therapy in critically ill adults. Nat Rev Nephrol. (2018) 14:541–57. 10.1038/s41581-018-0044-0 30072710

[25] Prowle JR Echeverri JE Ligabo EV Ronco C Bellomo R. Fluid balance and acute kidney injury. Nat Rev Nephrol. (2010) 6:107–15. 10.1038/nrneph.2009.213 20027192

[26] Perner A Prowle J Joannidis M Young P Hjortrup PB Pettilä V. Fluid management in acute kidney injury. Intensive Care Med. (2017) 43:807–15. 10.1007/s00134-017-4817-x 28470347

[27] Perner A Haase N Guttormsen AB Tenhunen J Klemenzson G Åneman A. Hydroxyethyl starch 130/0.42 versus ringer's acetate in severe sepsis. N Engl J Med. (2012) 367:124–34. 10.1056/NEJMoa1204242 22738085

[28] Wilcox CS. Regulation of renal blood flow by plasma chloride. J Clin Invest. (1983) 71:726–35. 10.1172/JCI110820 6826732 PMC436923

[29] Chowdhury AH Cox EF Francis ST Lobo DN. a randomized, controlled, double-blind crossover study on the effects of 2-l infusions of 0.9% saline and plasma-lyte® 148 on renal blood flow velocity and renal cortical tissue perfusion in healthy volunteers. Ann Surg. (2012) 256:18–24. 10.1097/SLA.0b013e318256be72 22580944

[30] Young P Bailey M Beasley R Henderson S Mackle D McArthur C. Effect of a buffered crystalloid solution vs saline on acute kidney injury among patients in the intensive care unit: the split randomized clinical trial. JAMA. (2015) 314:1701. 10.1001/jama.2015.12334 26444692

[31] Van Regenmortel N Verbrugghe W Roelant E Van den Wyngaert T Jorens PG. Maintenance fluid therapy and fluid creep impose more significant fluid, sodium, and chloride burdens than resuscitation fluids in critically ill patients: a retrospective study in a tertiary mixed ICU population. Intensive Care Med. (2018) 44:409–17. 10.1007/s00134-018-5147-3 29589054 PMC5924672

[32] McNab S Duke T South M Babl FE Lee KJ Arnup SJ. 140 mmol/L of sodium versus 77 mmol/L of sodium in maintenance intravenous fluid therapy for children in hospital (PIMS): a randomised controlled double-blind trial. The Lancet. (2015). 385:1190–7. 10.1016/S0140-6736(14)61459-8 25472864

[33] Moritz ML Ayus JC. Maintenance intravenous fluids in acutely ill patients. N Engl J Med. (2015) 373:1350–60. 10.1056/NEJMra1412877 26422725

[34] Lobo DN Stanga Z Simpson JA Anderson JA Rowlands BJ Allison SP. Dilution and redistribution effects of rapid 2-litre infusions of 0.9% (w/v) saline and 5% (w/v) dextrose on haematological parameters and serum biochemistry in normal subjects: a double-blind crossover study. Clin Sci Lond Engl. (2001). 101:173–9. 10.1042/cs1010173 11473492

[35] Van Regenmortel N De Weerdt T Van Craenenbroeck AH Roelant E Verbrugghe W Dams K. Effect of isotonic versus hypotonic maintenance fluid therapy on urine output, fluid balance, and electrolyte homeostasis: a crossover study in fasting adult volunteers. Br J Anaesth. (2017) 118:892–900. 10.1093/bja/aex118 28520883 PMC5455256

[36] Kashy BK Podolyak A Makarova N Dalton JE Sessler DI Kurz A. Effect of hydroxyethyl starch on postoperative kidney function in patients having noncardiac surgery. Anesthesiology. (2014) 121:730–9. 10.1097/ALN.0000000000000375 25054470 PMC4389778

[37] Serpa Neto A Veelo DP Peireira VGM de Assunção MSC Manetta JA Espósito DC. Fluid resuscitation with hydroxyethyl starches in patients with sepsis is associated with an increased incidence of acute kidney injury and use of renal replacement therapy: a systematic review and meta-analysis of the literature. J Crit Care. (2014) 29:185.e1-185.e7. 10.1016/j.jcrc.2013.09.031 24262273

[38] Yozova ID Howard J Adamik K-N. Retrospective evaluation of the effects of administration of tetrastarch (hydroxyethyl starch 130/0.4) on plasma creatinine concentration in dogs (2010–2013):201 dogs. J Vet Emerg Crit Care. (2016) 26:568–77. 10.1111/vec.12483 27144501

[39] Yozova ID Howard J Adamik KN. Effect of tetrastarch (hydroxyethyl starch 130/0.4) on plasma creatinine concentration in cats: a retrospective analysis (2010–2015). J Feline Med Surg. (2017) 19:1073–9. 10.1177/1098612X16676160 27803312 PMC11110996

[40] Sigrist NE Kälin N Dreyfus A. Changes in serum creatinine concentration and acute kidney injury (AKI) grade in dogs treated with hydroxyethyl starch 130/0.4 from 2013 to 2015. J Vet Intern Med. (2017) 31:434–41. 10.1111/jvim.14645 28109131 PMC5354072

[41] Hayes G Benedicenti L Mathews K. Retrospective cohort study on the incidence of acute kidney injury and death following hydroxyethyl starch (HES 10% 250/0.5/5:1) administration in dogs (2007–2010). J Vet Emerg Crit Care. (2016) 26:35–40. 10.1111/vec.12412 26587795

[42] Lewis SR Pritchard MW Evans DJ Butler AR Alderson P Smith AF. Colloids versus crystalloids for fluid resuscitation in critically ill people. Cochrane Database Syst Rev. (2018) 8:CD000567. 10.1002/14651858.CD000567.pub7 30073665 PMC6513027

[43] Rhodes A Evans LE Alhazzani W Levy MM Antonelli M Ferrer R. Surviving sepsis campaign: international guidelines for management of sepsis and septic shock: 2016. Intensive Care Med. (2017) 43:304–77. 10.1007/s00134-017-4683-6 28101605

[44] Wellman ML DiBartola SP Kohn CW. Chapter 1 - applied physiology of body fluids in dogs and cats. In: DiBartola SP, editor. Fluid, Electrolyte, and Acid-Base Disorders in Small Animal Practice. 4th Edn. Saint Louis, MO: W. B. Saunders (2012). p. 2–25. 10.1016/B978-1-4377-0654-3.00008-1

[45] Smith RC Haschem T Hamlin RL. Water and electrolyte intake and output and quantity of feces in the healthy dog. Vet Med Small Anim Clin. (1964) 59:743–8.

[46] O'Connor WJ Potts DJ. The external water exchanges of normal laboratory dogs. Q J Exp Physiol Cogn Med Sci. (1969) 54:244–65. 10.1113/expphysiol.1969.sp002022 5193737

[47] Hamlin R Tashjian R. Water and electrolyte intake and output and quantity of feces in healthy cats. Vet Med Small Anim Clin. (1964) 59:746–7.

[48] Thrall BE Miller LG. Water turnover in cats fed dry rations. Feline Pr. (1976) 6:10–7.464354

[49] Westgren F Ley CJ Kampa N Lord P. Effects of hydration on scintigraphic glomerular filtration rate measured using integral and plasma volume methods in dogs with suspected renal disease. Vet Radiol Ultrasound. (2014) 55:632–7. 10.1111/vru.12173 24837785

[50] Boscan P Pypendop BH Siao KT Francey T Dowers K Cowgill L. Fluid balance, glomerular filtration rate, and urine output in dogs anesthetized for an orthopedic surgical procedure. Am J Vet Res. (2010) 71:501–7. 10.2460/ajvr.71.5.501 20433374

[51] Brandstrup B Svendsen PE Rasmussen M Belhage B Rodt SÅ Hansen B. Which goal for fluid therapy during colorectal surgery is followed by the best outcome: near-maximal stroke volume or zero fluid balance? Br J Anaesth. (2012) 109:191–9. 10.1093/bja/aes163 22710266

[52] Cavanagh AA Sullivan LA Hansen BD. Retrospective evaluation of fluid overload and relationship to outcome in critically ill dogs: fluid overload in critically ill dogs. J Vet Emerg Crit Care. (2016) 26:578–86. 10.1111/vec.12477 27074594

[53] Hung S Lai Y Kuo K Tarng D. Volume overload and adverse outcomes in chronic kidney disease: clinical observational and animal studies. J Am Heart Assis. (2015) 4:e001918. 10.1161/JAHA.115.001918 25944876 PMC4599419

[54] Lim LM Tsai NC Lin MY Hwang DY Lin HYH Lee JJ. Hyponatremia is associated with fluid imbalance and adverse renal outcome in chronic kidney disease patients treated with diuretics. Sci Rep. (2016) 6:36817. 10.1038/srep36817 27841359 PMC5108044

[55] Tsai Y-C Tsai J-C Chen S-C Chiu Y-W Hwang S-J Hung C-C. Association of fluid overload with kidney disease progression in advanced CKD: a prospective cohort study. Am J Kidney Dis. (2014) 63:68–75. 10.1053/j.ajkd.2013.06.011 23896483

[56] Bouchard J Soroko SB Chertow GM Himmelfarb J Ikizler TA Paganini EP. Fluid accumulation, survival and recovery of kidney function in critically ill patients with acute kidney injury. Kidney Int. (2009) 76:422–7. 10.1038/ki.2009.159 19436332

[57] Woodward CW Lambert J Ortiz-Soriano V Li Y Ruiz-Conejo M Bissell BD. Fluid overload associates with major adverse kidney events in critically ill patients with acute kidney injury requiring continuous renal replacement therapy. Crit Care Med. (2019) 47:e753–60. 10.1097/CCM.0000000000003862 31162196

[58] Alobaidi R Morgan C Basu RK Stenson E Featherstone R Majumdar SR. Association between fluid balance and outcomes in critically ill children: a systematic review and meta-analysis. JAMA Pediatr. (2018) 172:257. 10.1001/jamapediatrics.2017.4540 29356810 PMC5885847

[59] Claure-Del Granado R Mehta RL. Fluid overload in the ICU: evaluation and management. BMC Nephrol. (2016) 17:109. 10.1186/s12882-016-0323-6 27484681 PMC4970195

[60] Cantarovich F Rangoonwala B Lorenz H Verho M Esnault VLM. High-dose furosemide for established ARF: a prospective, randomized, double-blind, placebo-controlled, multicenter trial. Am J Kidney Dis. (2004) 44:402–9. 10.1016/S0272-6386(04)00810-8 15332212

[61] Grams ME Estrella MM Coresh J Brower RG Liu KD. Fluid balance, diuretic use, and mortality in acute kidney injury. Clin J Am Soc Nephrol. (2011) 6:966–73. 10.2215/CJN.08781010 21393482 PMC3087792

[62] Uchino S Doig GS Bellomo R Morimatsu H Morgera S Schetz M. Diuretics and mortality in acute renal failure. Crit Care Med. (2004) 32:1669–77. 10.1097/01.CCM.0000132892.51063.2F 15286542

[63] Chawla LS Davison DL Brasha-Mitchell E Koyner JL Arthur JM Shaw AD. Development and standardization of a furosemide stress test to predict the severity of acute kidney injury. Crit Care. (2013) 17:R207. 10.1186/cc13015 24053972 PMC4057505

[64] Chen JJ Chang CH Huang YT Kuo G. Furosemide stress test as a predictive marker of acute kidney injury progression or renal replacement therapy: a systemic review and meta-analysis. Crit Care. (2020) 24:202. 10.1186/s13054-020-02912-8 32381019 PMC7206785

[65] Ng KT Velayit A Khoo DKY Mohd Ismail A Mansor M. Continuous infusion versus intermittent bolus injection of furosemide in critically ill patients: a systematic review and meta-analysis. J Cardiothorac Vasc Anesth. (2018) 32:2303–10. 10.1053/j.jvca.2018.01.004 29454528

[66] Jaber S Paugam C Futier E Lefrant J-Y Lasocki S Lescot T. Sodium bicarbonate therapy for patients with severe metabolic acidaemia in the intensive care unit (BICAR-ICU): a multicentre, open-label, randomised controlled, phase 3 trial. Lancet. (2018) 392:31–40. 29910040 10.1016/S0140-6736(18)31080-8

[67] Zhang Z Zhu C Mo L Hong Y. Effectiveness of sodium bicarbonate infusion on mortality in septic patients with metabolic acidosis. Intensive Care Med. (2018) 44:1888–95. 10.1007/s00134-018-5379-2 30255318

[68] Brown N Segev G Francey T Kass P Cowgill LD. Glomerular filtration rate, urine production, and fractional clearance of electrolytes in acute kidney injury in dogs and their association with survival. J Vet Intern Med. (2015) 29:28–34. 10.1111/jvim.12518 25594609 PMC4858109

